# Early insulin resistance in normoglycemic low-risk individuals is associated with subclinical atherosclerosis

**DOI:** 10.1186/s12933-023-02090-1

**Published:** 2023-12-19

**Authors:** Josep Iglesies-Grau, Ana Garcia-Alvarez, Belén Oliva, Guiomar Mendieta, Inés García-Lunar, José J. Fuster, Ana Devesa, Cristina Pérez-Herreras, Antonio Fernández-Ortiz, Ramon Brugada, Borja Ibanez, Rodrigo Fernandez-Jimenez, Valentin Fuster

**Affiliations:** 1 Research Center and Centre ÉPIC, Montreal Heart Institute, Montréal, Canada; 2 Universitat de Girona, Girona, Spain; 3 Centro Nacional de Investigaciones Cardiovasculares (CNIC), Calle Melchor Fernández Almagro, 3, Madrid, 28029 Spain; 4 Cardiology Departement, Institut Clinic Cardiovascular, Hospital Clínic de Barcelona, Barcelona, Spain; 5 Institut d’Investigacions Biomèdiques August Pi i Sunyer (IDIBAPS), Barcelona, Spain; 6 CIBER de Enfermedades Cardiovasculares (CIBERCV), Madrid, Spain; 7 Cardiology Department, University Hospital La Moraleja, Madrid, Spain; 8 The Zena and Michael A. Wiener Cardiovascular Institute, Icahn School of Medicine at Mount Sinai (ISMMS), New York, NY USA; 9 Medical Services, Banco Santander, Madrid, Spain; 10 Hospital Universitario Clínico San Carlos, Madrid, Spain; 11 Department of Cardiology, Hospital Universitari Dr. Josep Trueta, Girona, Spain; 12 Institut d’Investigació Biomèdica de Girona (IDIBGI), Girona, Spain; 13 IIS-Fundación Jiménez Díaz University Hospital, Madrid, Spain

**Keywords:** Imaging, Insulin resistance, Atherosclerosis, Cardiovascular disease, Primary disease prevention

## Abstract

**Background:**

Elevated glycated hemoglobin (HbA1c) is associated with a higher burden of subclinical atherosclerosis (SA). However, the association with SA of earlier insulin resistance markers is poorly understood. The study assessed the association between the homeostatic model assessment of insulin resistance index (HOMA-IR) and SA in addition to the effect of cardiovascular risk factors (CVRFs) in individuals with normal HbA1c.

**Methods:**

A cohort of 3,741 middle-aged individuals from the Progression of Early Subclinical Atherosclerosis (PESA) study with basal HbA1c < 6.0% (< 42 mmol/mol) and no known CV disease underwent extensive imaging (multiterritorial vascular ultrasound and coronary artery calcium score, CACS) to assess the presence, burden, and extent of SA.

**Results:**

Individuals with higher HOMA-IR values had higher rates of CVRFs. HOMA-IR showed a direct association with the multiterritorial extent of SA and CACS (p < 0.001) and with global plaque volume measured by 3-dimensional vascular ultrasound (p < 0.001). After adjusting for key CVRFs and HbA1c, HOMA-IR values ≥ 3 were associated with both the multiterritorial extent of SA (odds ratio 1.41; 95%CI: 1.01 to 1.95, p = 0.041) and CACS > 0 (odds ratio 1.74; 95%CI: 1.20 to 2.54, p = 0.004), as compared with the HOMA-IR < 2 (the reference HOMA-IR category). In a stratified analysis, this association remained significant in individuals with a low-to-moderate SCORE2 risk estimate (75.6% of the cohort) but not in high-risk individuals.

**Conclusions:**

The use of HOMA-IR identified low-risk individuals with a higher burden of SA, after adjusting for the effects of key traditional CVRFs and HbA1c. HOMA-IR is a simple measure that could facilitate earlier implementation of primary CV prevention strategies in clinical practice.

**Supplementary Information:**

The online version contains supplementary material available at 10.1186/s12933-023-02090-1.

## Introduction

Cardiovascular disease (CVD) is preceded by a slow, progressive subclinical disease that can start as early as childhood. Similarly, early insulin resistance, defined as the slow progressive need for higher fasting insulin to maintain normal fasting glucose concentrations, can be seen years or decades before the appearance of increased fasting glucose, glucose intolerance, and elevated glycated hemoglobin (HbA1c) that are characteristic of the insulin resistance–prediabetes–type 2 diabetes spectrum [1].

Elevated HbA1c is an established index of cumulative exposure to high blood glucose that has already been shown to be associated with subclinical atherosclerosis (SA), even at values below the thresholds for prediabetes and type 2 diabetes. Moreover, combining HbA1c with classical cardiovascular risk factors (CVRFs) significantly improves the prediction of the multiterritorial extent of SA [2]. However, there is no previously published information about the potential added value of identifying earlier stages of insulin resistance when HbA1c is still “normal” or the association with the presence of SA. Insulin is rarely measured in routine clinical practice, precluding identification of early insulin resistance until later, more advanced stages of the type 2 diabetes disease spectrum. Similarly, SA frequently goes undetected until the first clinical event.

The homeostatic model assessment of insulin resistance (HOMA-IR) ratio is an easy-to-measure surrogate of early insulin resistance. The higher the insulin concentration needed to maintain low fasting glucose levels, the higher the HOMA-IR value, indicating the early development of insulin resistance [3]. HOMA-IR also has the advantage of being a simple, feasible, and economical measure that can be easily integrated into medical practice and is a proven robust clinical and epidemiological tool [4]. The present study explored the association of HOMA-IR with SA in a large cohort of middle-aged individuals with normal HbA1c and no established CVD.

## Methods

### Study overview and population

The PESA (Progression of Early Subclinical Atherosclerosis)-CNIC-Santander study (NCT01410318) uses extensive noninvasive vascular imaging modalities to characterize the presence and progression of SA in a prospective cohort of 4,184 middle-aged asymptomatic employees of Santander Bank in Madrid. The adult participants were between 40 and 55 years old at recruitment and were prospectively included if the baseline examination showed no cardiovascular or chronic kidney disease or any other condition that might reduce life expectancy or affect study adherence. The Instituto de Salud Carlos III Ethics Committee approved the study protocol, and all eligible participants provided written informed consent. The study rationale, design, and data collection details have been described elsewhere [5].

For the present study, 367 participants with prediabetes, type 2 diabetes, or under hypoglycemic treatment at baseline were excluded. Type 2 diabetes was defined as fasting plasma glucose ≥ 126 mg/dL, baseline HbA1c ≥ 6.5% (≥ 48 mmol/mol), or treatment with insulin or oral hypoglycemic medication. Prediabetes was defined according to National Institute for Health and Care Excellence [NICE] guidelines as baseline HbA1c between 6.0% (42 mmol/mol) and 6.4% (46 mmol/mol). Another 76 participants were excluded due to incomplete imaging test information (carotid, femoral, aorta, or CACS). The final sample for the present analysis, therefore, included 3,741 participants.

### Variables collected

CVRFs, other than prediabetes and diabetes, were determined from blood samples, study visits, and interviews. (1) Systemic arterial hypertension: systolic blood pressure (SBP) ≥ 140 mm Hg, diastolic blood pressure (DBP) ≥ 90 mm Hg, or use of antihypertensive medication; (2) Dyslipidemia: total cholesterol ≥ 240 mg/dL, low-density lipoprotein cholesterol ≥ 160 mg/dL, high-density lipoprotein cholesterol (HDL-C) < 40 mg/dL, or use of lipid-lowering drugs; (3) Smoking: current smoking status and lifetime consumption of > 100 cigarettes; (4) Sedentary lifestyle: reporting > 8 h per day in a sitting position; (5) CVD family history: having a first-degree relative diagnosed with atherosclerosis below the age of 55 years in men and 65 years in women; (6) Metabolic syndrome, defined according to the modified ATP III Criteria as meeting at least three of the following conditions: central obesity (waist circumference ≥ 88 cm in women and ≥ 102 cm in men), elevated plasma triglycerides (≥ 150 mg/dL), low plasma HDL-C (< 40 mg/dL in men or < 50 mg/dL in women), elevated fasting plasma glucose (≥ 100 mg/dL), and high blood pressure (SBP ≥ 130 mmHg and/or DBP ≥ 85 mmHg).

To study the association between HOMA-IR and biochemical evidence of hepatic steatosis, the non-alcoholic fatty liver disease (NAFLD) score was calculated for each participant based on routinely available clinical data and three serological markers: fasting insulin, alanine transaminase (ALT), and aspartate transaminase (ALT) [6]. The presence of hepatic steatosis was defined as predicted intrahepatic fat ≥ 5% of liver weight [7].

### HOMA-IR categories

Basal insulin resistance was calculated using the original HOMA-IR formula: HOMA-IR = fasting insulin (mU/L) x fasting plasma glucose (mg/dL) / 405 [3]. Based on previous evidence indicating that metabolic risk increases for values ≥ 2, HOMA-IR values were grouped into three categories, chosen to study associations and dose-response effects while maintaining clinical meaningfulness [8]: <2 (reference category), 2 to < 3, and ≥ 3.

### Estimating participant CV risk

The 10-year risk of first-onset CVD (fatal and non-fatal) was calculated for each participant using the updated regional and sex-specific SCORE2 risk prediction models for 10-year risk in European populations. As all participants were employees of Santander Bank in Madrid, Spain, the equation for populations with a low CVD risk was selected [9]. This equation includes the following parameters: age (in years), sex, measured SBP (mmHg), smoking status, measured total cholesterol (mg/dL), and measured HDL-C (mg/dL). Based on the SCORE2 risk and using different numerical cutoffs depending on age group (< 50 years and 50–69 years), 10-year CVD risk was reclassified into two categories: low-to-moderate CVD risk (< 2.5% for participants < 50 years and < 5% for those ≥ 50 years) and high CVD risk (≥ 2.5 to 7.5% for participants < 50 years and ≥ 5–10% for those ≥ 50 years). Three other CV risk scores were also calculated: the 10-year Framingham Risk Score [10], the 30-year Framingham Risk Score [11], and the Regicor Risk Score [12].

### Imaging protocol, analyses, and definition of atherosclerosis

The presence and extent of SA were evaluated using three imaging modalities: (a) 2-dimensional ultrasound (2DVUS) cross-sectional sweeps were made of the carotid, abdominal aortic, and femoral territories; (b) the coronary artery calcification score (CACS) was obtained by non-contrast computed tomography using the Agatston method; and (c) 3-dimensional vascular ultrasound (3DVUS) with standardized 6-cm acquisition was used to quantify atheroma plaque volume in the carotid and femoral arteries. The presence of plaque by 2DVUS was defined according to the Mannheim consensus. Plaque was defined as a focal structure encroaching into the arterial lumen, measuring ≥ 0.5 mm or greater than 50% of the surrounding intima-media thickness. Alternatively, it was considered as plaque if it exhibited a diffuse thickness ≥ 1.5 mm, measured from the media–adventitia to the intima–lumen interface in any of the territories analyzed. Complete PESA study imaging protocol details have been reported elsewhere [5, 13].

A total of 6 vascular territories were defined: right carotid, left carotid, abdominal aorta, right femoral, left femoral, and the coronary arteries. The extent of atherosclerosis was defined by the number of vascular territories showing the presence of plaque identified by 2DVUS or CACS ≥ 1 in the case of the coronary arteries (disease-free if 0 vascular sites affected; multiterritorial extent of disease if ≥ 1 territory was affected). Global plaque volume (mm3) was defined as the sum of right and left 3DVUS-determined plaque volumes in each vascular territory as previously described [14]. All ultrasound recordings and digital images were analyzed at the CNIC Core Imaging Laboratory by experienced evaluators blinded to participant data. Interoperator reproducibility assessments have been reported elsewhere [13].

### Statistical analysis

The distribution of continuous variables was assessed with graphical methods. Summary statistics describing baseline characteristics are presented as means ± standard deviation (SD) or median and [interquartile range] as appropriate for continuous variables and as count and frequencies for categorical variables. Crude pairwise comparisons among HOMA-IR category groups were performed by Student’s t-test or Wilcoxon signed-rank test and χ2 or Fisher exact test, for continuous and categorical variables, as appropriate, and differences were considered statistically significant in these cases at p-value < 0.025 = (0.05/ number of comparisons).Trend tests among HOMA-IR categories were performed by linear or ordered/binary logistic regression as appropriate. For multivariable models, covariates were selected according to their reported association with atherosclerosis (clinical plausibility) [2]. Thus, estimates were adjusted for age, sex, smoking status, SBP, DBP, low-density lipoprotein cholesterol (LDL-C), HDL-C, body mass index (BMI), family history of CVD, and HbA1c. Statistical analyses were performed with STATA (2017, Stata Statistical Software: Release 17.0, StataCorp, College Station, TX, USA).

## Results

### Study population

The study included 3,741 CVD-free participants without prediabetes or type 2 diabetes and with complete imaging test information (89.4% of the total PESA cohort). The mean participant age was 45.5 ± 4.2 years, and 38.7% were women. Median HbA1c was 5.4% [5.2–5.6%] or 36 mmol/mol [33 to 38]. The median HOMA-IR value was 1.08 [0.74 to 1.60], and in most participants (~ 85.0%) HOMA-IR was < 2 (reference), in ~ 11% between 2 and 3, and in ~ 4% ≥3. The full distribution of HOMA-IR values is shown in Supplementary Fig. 1. Observed correlation between HbA1c and HOMA-IR values was weak (Spearman’s *rho* = 0.14; 95%CI: 0.11–0.17). The median 10-year risk of combined fatal and nonfatal CVD events, according to the SCORE2 risk equation was 2.0% [1.2–3.1%]. Most participants (~ 75.6%) were classified as having low-to-moderate CVD risk, the rest being at high CVD risk, with none at very-high CVD risk. The baseline characteristics of the study participants are summarized in Table 1. Baseline characteristics according to risk category are presented in Supplementary Tables 1 and 2.

**Table 1:** Baseline study population clinical characteristics (N = 3,741) stratified by HOMA-IR category

	Total population (N = 3,741)	HOMA-IR < 2 (reference) (n = 3,181)	HOMA-IR 2 to < 3 (n = 410)	HOMA-IR ≥ 3 (n = 150)	P value
Demographic					
Age, years	45.5 ± 4.2	45.4 ± 4.2	46.4 ± 4.3	46.9 ± 4.2	< 0.001^a,b^
Sex, male	2,295 (61.3)	1,815 (57.1)	349 (85.1)	131 (87.3)	< 0.001^a,b^
Race/ethnicity, Caucasian	3,736 (99.9)	3,176 (99.8)	410 (100)	150 (100)	0.808
**Risk factors**					
Hypertension	389 (10.4)	244 (7.7)	95 (23.2)	50 (33.3)	< 0.001^a,b^
Dyslipidemia	1,468 (39.2)	1,096 (34.4)	271 (66.1)	101 (67.3)	< 0.001^a,b^
Smoking	743 (20.0)	638 (20.2)	82 (20.3)	23 (15.3)	0.190
Sedentary lifestyle	3,642 (98.5)	3,099 (98.6)	395 (97.8)	148 (99.3)	0.875
Family history of CV disease	585 (15.6)	474 (14.9)	79 (19.3)	32 (21.3)	< 0.001^a^
Metabolic syndrome	279 (7.5)	94 (3.0)	111 (27.1)	74 (49.3)	< 0.001^a,b^
**Measured risk and health factors**					
SBP, mmHg	115.7 ± 12.5	114.3 ± 12.0	123.0 ± 12.3	125.3 ± 11.8	< 0.001^a,b^
DBP, mmHg	72.1 ± 9.4	71.0 ± 8.9	78.2 ± 9.2	79.6 ± 9.3	< 0.001^a,b^
Total cholesterol, mg/dL	199.8 ± 32.7	198.0 ± 32.0	209.3 ± 35.1	212.2 ± 32.3	< 0.001^a,b^
HDL-C, mg/dL	49.5 ± 12.2	50.8 ± 12.1	42.3 ± 9.4	40.2 ± 8.7	< 0.001^a,b^
LDL-C, mg/dL	131.9 ± 29.2	130.3 ± 28.8	141.2 ± 30.1	140.0 ± 27.6	< 0.001^a,b^
Non-HDL-C, mg/dL	150.4 ± 33.1	147.2 ± 32.1	167.0 ± 33.6	172.0 ± 31.6	< 0.001^a,b^
Triglycerides, mg/dL	78 [59–109]	73 [56–99]	117 [85–151]	132 [103–197]	< 0.001^a,b^
BMI, kg/m^2^	25.9 ± 3.6	25.2 ± 3.2	29.3 ± 3.2	30.9 ± 3.6	< 0.001^a,b^
Waist, cm	88.5 ± 11.5	86.4 ± 10.5	99.4 ± 9.1	103.5 ± 9.3	< 0.001^a,b^
Basal Insulin, mU/L	5 [3.5–7.1]	4.5 [3.3-6.0]	10.1 [9.3–11]	14.8 [13.4–17.6]	< 0.001^a,b^
Basal glucose, mg/dL	88 [83–94]	87 [82–93]	95 [90–100]	99 [94–104]	< 0.001^a,b^
HbA1c, %	5.4 [5.2–5.6]	5.4 [5.1–5.6]	5.4 [5.2–5.7]	5.5 [5.4–5.7]	< 0.001^a,b^
Ratio total-cholesterol/HDL	4.1 [3.4–4.9]	3.9 [3.3–4.7]	5.0 [4.2–5.8]	5.3 [4.6–6.4]	< 0.001^a,b^
Ratio triglycerides/HDL	1.6 [1.1–2.5]	1.5 [1.0-2.2]	2.7 [1.9-4.0]	3.6 [2.4–5.2]	< 0.001^a,b^
TyG index	4.4 [4.3–4.6]	4.4 [4.2–4.5]	4.6 [4.5–4.8]	4.7 [4.6–4.9]	< 0.001^a,b^
Mod/vig activity, min/week	241 [160–346]	244 [162–349]	233 [152–335]	223 [142–357]	< 0.001
**Inflammatory markers**					
hs-CRP, mg/dL	0.09 [0.05–0.18]	0.08 [0.04–0.16]	0.15 [0.09–0.28]	0.16 [0.10–0.30]	< 0.001^a,b^
Ferritin, ng/mL	55.4 [25.0-126.0]	50.5 [23.6-110.7]	135.1 [57.2-213.3]	125.4 [46.6-221.2]	< 0.001^a,b^
1–hour ESR, mm	5 [4–8]	5 [4–8]	5 [4–8]	6 [4–9]	0.572
Fibrinogen, mg/dL	258.6 [231.9-288.7]	257.5 [231.4-287.7]	262.6 [233.7-296.4]	268.5 [248.6-301.5]	< 0.001^a,b^
**Risk scores**					
SCORE2, %	2.0 [1.2–3.1]	1.9 [1.1–2.9]	2.8 [2.1–3.9]	3.1 [2.4–4.1]	< 0.001^a,b^
Framingham risk score 10y, %	5.5 [3.4-9.0]	5.1 [3.1–8.2]	8.5 [6.0-13.2]	10.7 [7.1–14.4]	< 0.001^a,b^
Framingham risk score 30y, %	14.4 [8.4–22.5]	13.0 [7.7–20.7]	22.1 [15.5–31.8]	26.0 [18.8–33.3]	< 0.001^a,b^
Regicor risk score, %	1.6 [0.9–2.5]	1.4 [0.8–2.3]	2.5 [1.7–3.5]	2.9 [2.1–3.9]	< 0.001^a,b^

Values are mean ± SD, n (%), or median [first quartile, third quartile]. Indicated p-value derived from trend tests among HOMA-IR categories

*HOMA-IR* homeostatic model assessment of insulin resistance index, *CV* cardiovascular, *SBP* systolic blood pressure, *DBP* diastolic blood pressure, *HDL-C* high-density lipoprotein cholesterol, *LDL-C* low-density lipoprotein cholesterol, *BMI* body mass index, *HbA1c* glycated hemoglobin, *TyG* triglyceride to glucose, *Mod/Vig activity min/week* moderate to vigorous minutes of physical activity per week, *hs-CRP* high-sensitivity C-reactive protein, *1-hour ESR* erythrocyte sedimentation rate, *SCORE2* systematic coronary risk estimation 2

^a^Indicates statistically significant differences (p < 0.025) between HOMA-IR < 2 and HOMA-IR 2 to < 3 groups

^b^Indicates statistically significant differences (p < 0.025) between HOMA-IR < 2 and HOMA-IR ≥ 3 groups

The participant groups with HOMA-IR > 2 had significantly higher rates of hypertension, dyslipidemia, a family history of CV disease, and metabolic syndrome (p < 0.001) and had higher CVRF measures, including for SBP, DBP, body mass index, waist circumference, as well as indicators of poorer metabolic health, including values for total cholesterol, HDL-C, LDL-C, non-HDL-C, plasma triglycerides, HbA1c, and inflammatory parameters (p < 0.001). These participants also had higher liver enzymes, a higher calculated NAFLD score, and a higher proportion of hepatic steatosis (p < 0.001, Supplementary Table 3).

### Association between HOMA-IR category and subclinical atherosclerosis

**Fig. 1:**
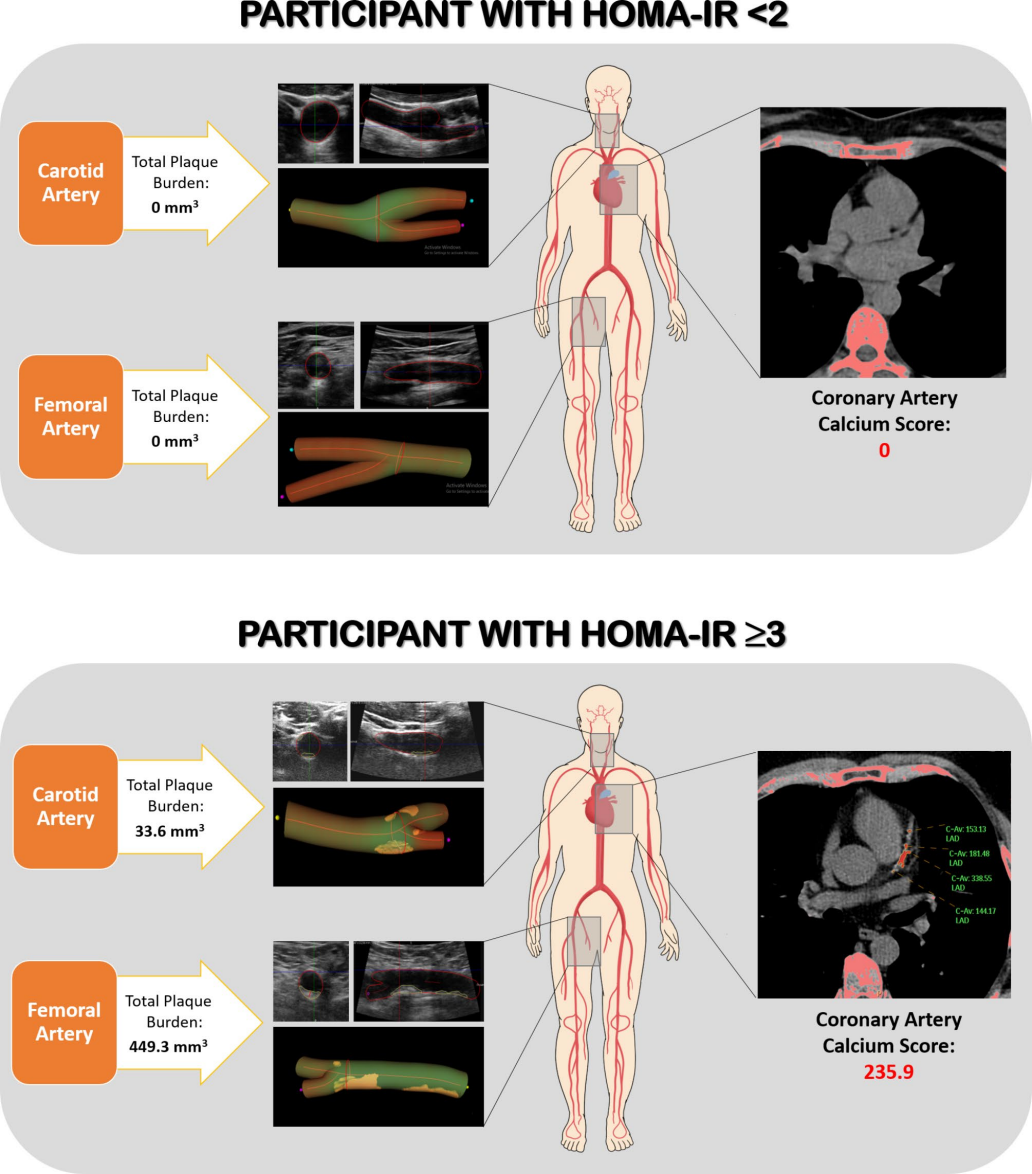
3-dimensional vascular ultrasound and computed tomography images exemplifying the study findings. (**A**) Visual example illustrating imaging findings in a participant with HOMA-IR < 2 (reference group), depicting the absence of subclinical atherosclerosis affecting vascular sites. (**B**) Visual example illustrating imaging findings in a participant with HOMA-IR ≥ 3 (category with the highest HOMA-IR values), revealing a multiterritorial extent of subclinical atherosclerosis in the carotid and femoral arteries and an elevated coronary artery calcium score. HOMA-IR = homeostatic model assessment of insulin resistance

**Fig. 2:**
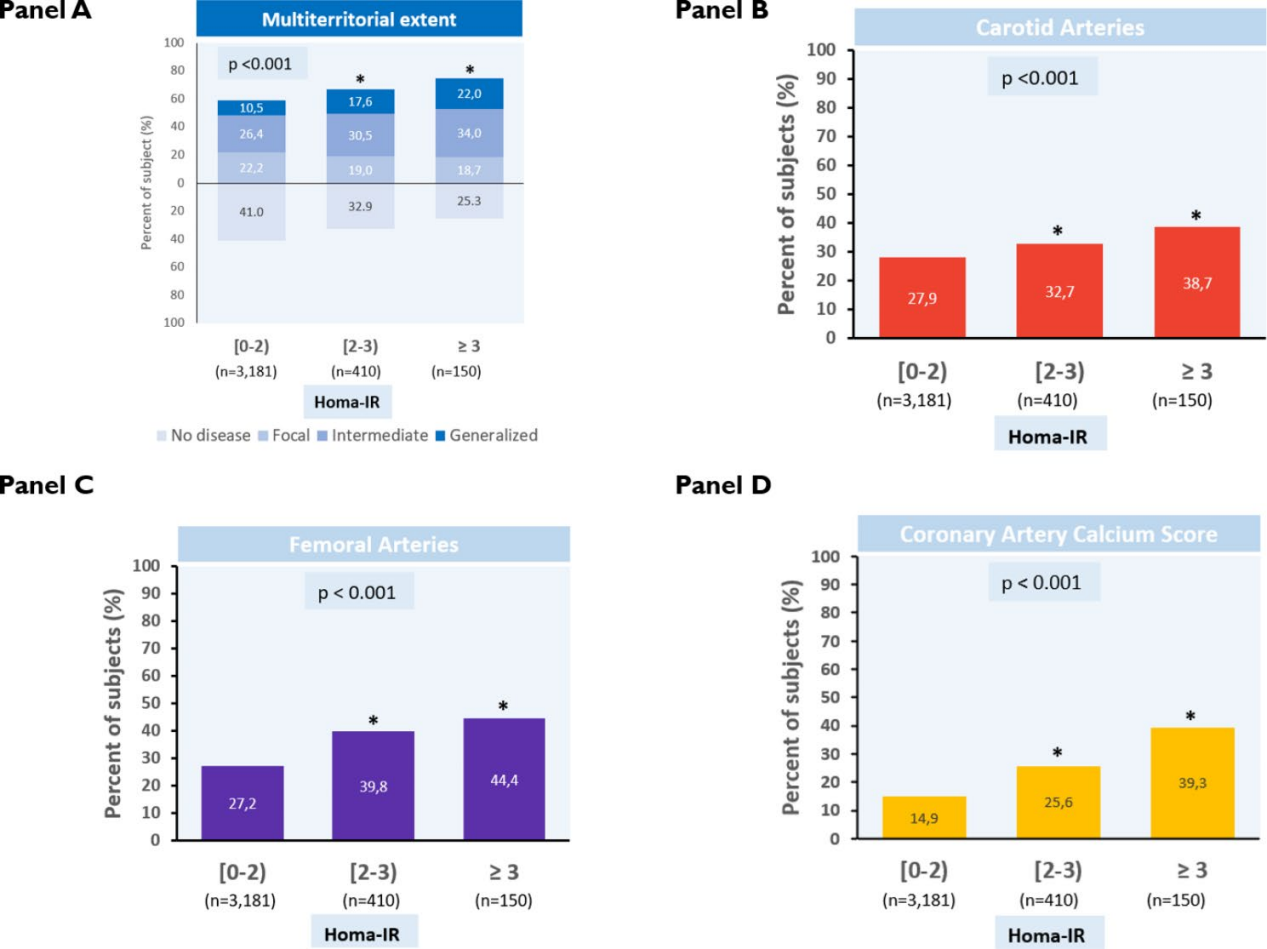
HOMA-IR categories, and association with the multiterritorial extent of subclinical atherosclerosis in different vascular territories. (**A**) Multiterritorial extent of subclinical atherosclerosis (SA), assessed by 2-dimensional vascular ultrasound and non-contrast coronary computed tomography, stratified by HOMA-IR category. Multiterritorial SA extent for this figure is defined by combining data from both imaging techniques to classify individuals as having no disease (0 vascular sites affected) or having focal (1 site), intermediate (2 to 3 sites), or generalized atherosclerosis (4 to 6 sites). (**B**–**D**) Presence of SA in different vascular territories stratified by HOMA-IR category. *Indicates statistically significant differences (p < 0.025) as compared to the lowest HOMA-IR category (HOMA-IR < 2, reference group). HOMA-IR = homeostatic model assessment of insulin resistance

Figure 1 provides visual examples illustrating the association between HOMA-IR and imaging findings of subclinical atherosclerosis. High HOMA-IR values showed a positive association with the multiterritorial extent of SA assessed by 2D vascular ultrasound and non-contrast cardiac computed tomography (p < 0.001) (Fig. 2A). This trend held for three individual territories: the carotid, femoral, and coronary (CACS) (Fig. 2B–D). In a sensitivity analysis, the association was also observed in individuals with HbA1c below and greater than or equal to the median value of 5.4% (36 mmol/mol) (p < 0.001) (Supplementary Figs. 2 and 3). Similar trends were observed when categorizing HOMA-IR values by tertiles, with significant differences found between the lowest and the highest tertile of HOMA-IR values (HOMA-IR < 0.8 and HOMA-IR ≥ 1.4, respectively) for all outcomes analyzed (data not shown).

In the multivariable regression analyses after adjusting for key CVRFs and HbA1c, the higher HOMA-IR categories maintained an association both with multiterritorial SA extent (odds ratio for HOMA-IR ≥ 3 = 1.41 [95%CI: 1.01 to 1.95, p = 0.041]) and with CACS (odds ratio for HOMA-IR ≥ 3 = 1.74 [95CI: 1.20 to 2.54, p = 0.004)], as compared with the HOMA-IR < 2 (reference category).

**Fig. 3:**
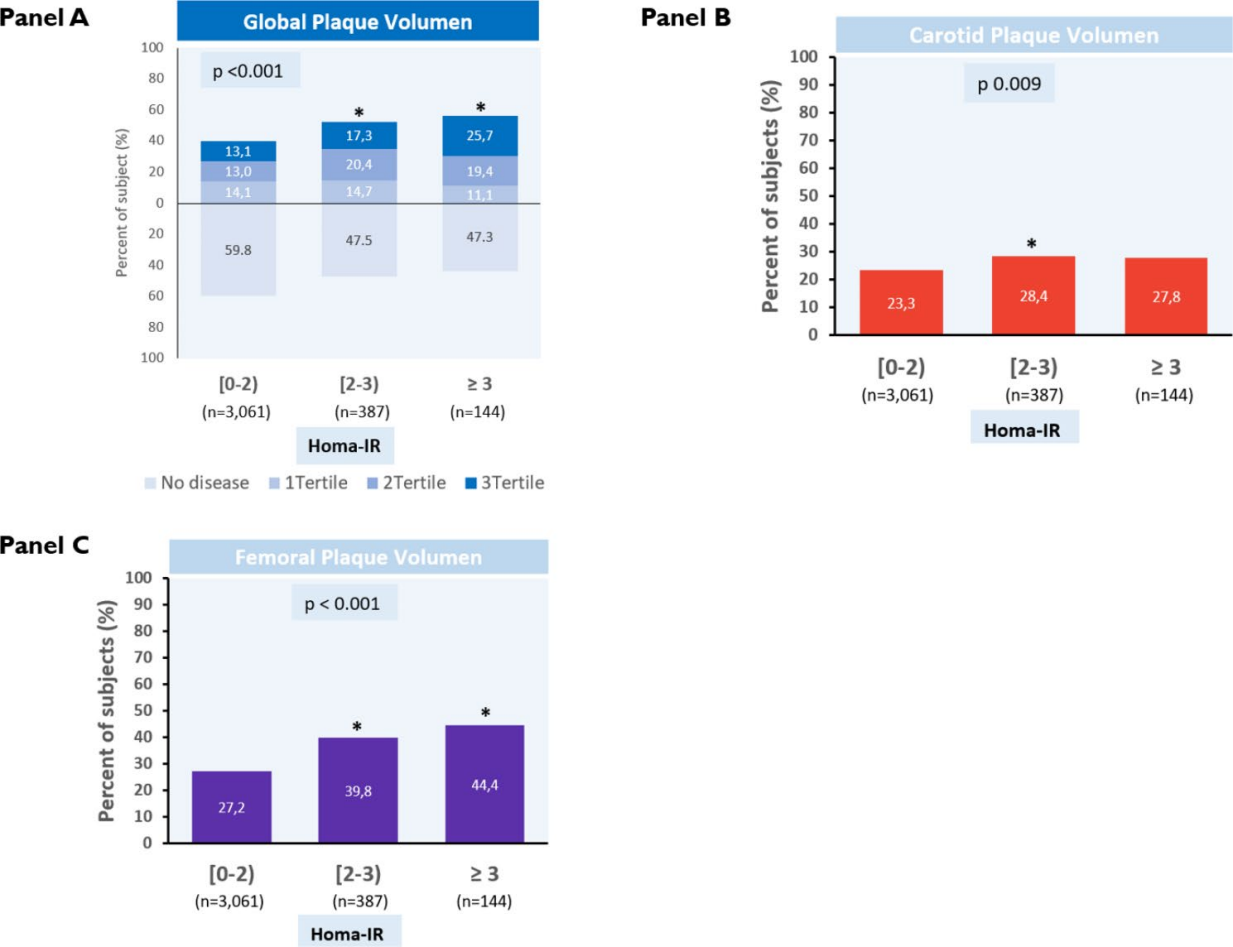
HOMA-IR categories and plaque volume measured by 3-dimensional vascular ultrasound (3DVUS). (**A**) Global plaque volume of subclinical atherosclerosis (no disease vs. volume in tertiles) assessed by 3DVUS and stratified by HOMA-IR category. (**B**, **C**) The presence of carotid and femoral plaque was assessed by 3DVUS (plaque volume > 0 mm^3^) and stratified by the HOMA-IR category. *Indicates statistically significant differences as compared to the lowest HOMA-IR category (HOMA-IR < 2, reference group)

Of the 3,741 participants, 3,592 (~ 96.0%) underwent 3DVUS imaging. After adjusting for key CVRFs and HbA1c, higher HOMA-IR categories showed a significant association with elevated global plaque burden (p < 0.001) and with the presence of SA in the carotid (p = 0.009) and femoral arteries (p < 0.001) when analyzed separately (Fig. 3A–C). Sensitivity analysis, including further adjustment for non-HDL cholesterol and high-sensitivity C-reactive protein (hs-CRP), showed similar results (data not shown).

### Associations between SCORE2 risk, HOMA-IR, and SA

**Fig. 4:**
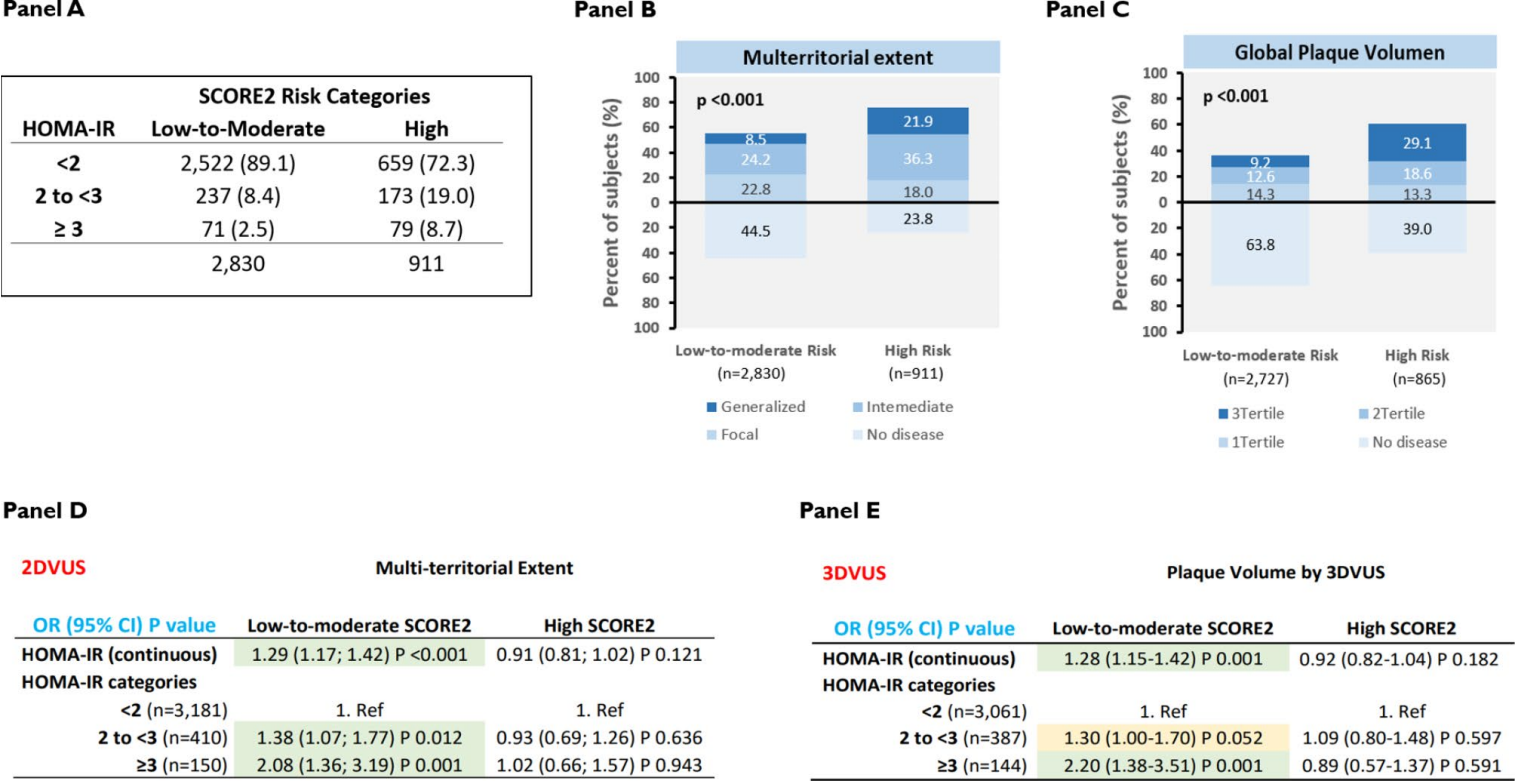
HOMA-IR, multiterritorial extent of subclinical atherosclerosis, and global plaque volume, stratified by SCORE2 risk category. (**A**) Number of participants in each SCORE2 risk category, stratified by HOMA-IR category. (**B**) Extent of subclinical atherosclerosis stratified by SCORE2 risk category. (**C**) Global plaque volume (absence of disease or plaque volume in tertiles) stratified by SCORE2 risk category. (**D**) SCORE2 risk-adjusted odds ratios for the multiterritorial extent of subclinical atherosclerosis stratified by HOMA-IR category. (**E**) Adjusted odds ratios for global plaque volume (absence of disease or plaque volume in tertiles) stratified by HOMA-IR category and SCORE2 risk category. The multivariable models were adjusted for age, sex, smoking status, systolic blood pressure, diastolic blood pressure, low-density lipoprotein cholesterol, high-density lipoprotein cholesterol, body mass index, family history of CVD, and HbA1c. OR = odds ratio, SCORE2 = systematic coronary risk evaluation score 2, HOMA-IR = homeostatic model assessment of insulin resistance, 2DVUS = 2-dimensional vascular ultrasound, 3DVUS = 3-dimensional vascular ultrasound

Stratification of participants by SCORE2 risk category revealed that individuals with high CVD risk had higher HOMA-IR values, a greater multiterritorial extent of SA (p < 0.001), and a higher global plaque volume (p < 0.001) than individuals with low-to-moderate CVD risk (Fig. 4A–C). SCORE2 risk stratification of the adjusted association between HOMA-IR categories, the multiterritorial extent of SA, and global plaque volume revealed an independent association between higher HOMA-IR and SA burden in low-to-moderate risk participants, but this association was not observed in high-risk individuals (Fig. 4D, E).

## Discussion

To our knowledge, this cross-sectional analysis of the PESA cohort study is the first to report an independent association between HOMA-IR-estimated early insulin resistance and SA measured by 2D vascular ultrasound, non-contrast cardiac computed tomography, and 3D vascular ultrasound in CVD-free individuals without prediabetes or type 2 diabetes (HbA1c < 6.0%, < 42 mmol/mol).

The assessment of the HOMA-IR ratio in this population with normal HbA1c conferred added value in at least 3 respects. First, it identified individuals with higher measured values for CVRFs, including SBP, DBP, body mass index, and waist circumference, as well as indicators of poorer metabolic status, including values for HbA1c, lipid profile, inflammatory parameters, liver parameters, and hepatic steatosis. Second, it identified a group of individuals with an elevated prevalence and extent of SA in the carotid, femoral, and coronary arterial beds, as well as a higher overall disease burden quantified by 3DVUS global plaque volume; notably, this association was found regardless of whether HbA1c was above or below the median value of 5.4% (36 mmol/mol) (p < 0.001). Finally, after adjusting for CVRFs and HbA1c, the odds of multiterritorial SA and CACS were higher among participants with HOMA-IR ≥ 3, particularly those with a low-to-moderate SCORE2 CVD risk estimate. Moreover, these odds decreased with the HOMA-IR category, being lower for participants with HOMA-IR = 2–3 and lowest for those with HOMA-IR ≤ 2.

**Fig. 5:**
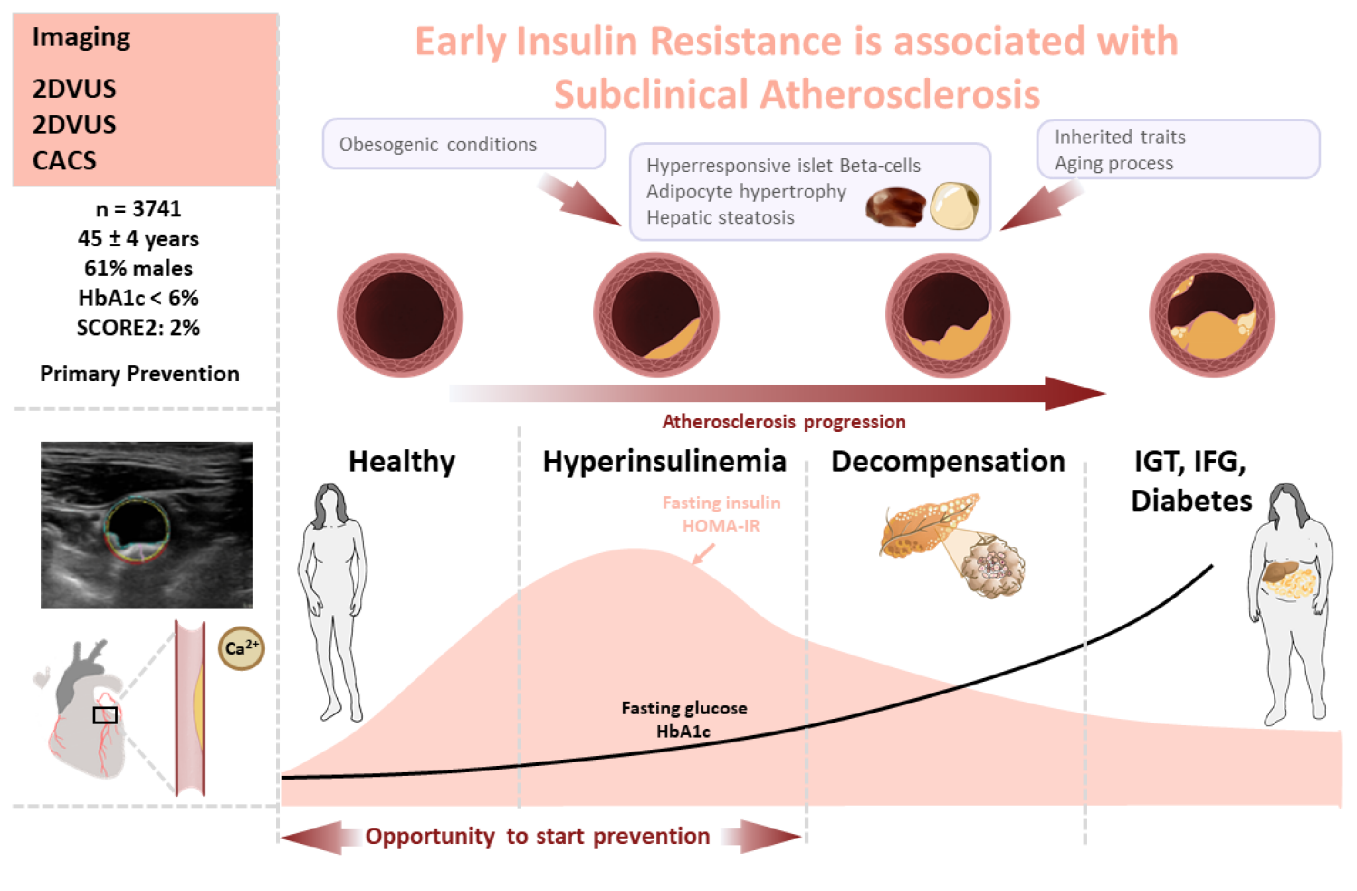
**Central Illustration**: Early insulin resistance and subclinical atherosclerosis. This study examined 3,741 individuals with normal HbA1c < 6.0% <42 mmol/mol) and no known cardiovascular disease. Higher homeostatic model assessment of insulin resistance (HOMA-IR) values were associated with higher subclinical atherosclerosis (SA) burden as measured by extensive imaging after adjusting for traditional CVRFs and HbA1c. The HOMA-IR index offers a simple and cost-effective way to detect the onset of insulin resistance and SA and could be used to support the implementation of early prevention strategies in clinical practice. 2DVUS, 2-dimensional vascular ultrasound; 3DVUS, 3-dimensional vascular ultrasound; CACS, coronary artery calcium score; IGT, impaired glucose tolerance; IFG, impaired fasting glucose

Our findings have important implications, considering that HOMA-IR is a simple clinical parameter that identifies the onset of insulin resistance before the rise of HbA1c and only requires a regular blood test. HOMA-IR screening could be used to facilitate early identification of individuals with a high risk of SA and help refine and further individualize primary prevention measures. By serving as an early indicator of susceptibility to the development of advanced atherosclerosis, HOMA-IR screening could prompt the early implementation of prevention strategies, particularly among individuals with low-to-moderate CVD risk (Fig. 5- Central Illustration).

### Value of HOMA-IR as an early marker of poorer metabolic health

Prediabetes and type 2 diabetes are advanced stages of the dysglycemia-based chronic disease continuum associated with an increased risk of CVD [1, 15]. Yet both the development of SA and frequently the first clinical events, occur earlier and at HbA1c levels below the diagnostic thresholds [16–18]. The question thus remains of whether we are properly addressing primary prevention of CVD by only screening for and treating diabetes and hyperlipidemia while ignoring the early onset of insulin resistance.

The HOMA-IR ratio captures early abnormalities in systemic glucose homeostasis by testing how much insulin is needed to keep fasting glycemia low. For instance, a fasting plasma insulin of 5 mU/L is needed for a healthy individual to maintain fasting plasma glucose at 81 mg/dL, and this results in HOMA-IR = 1 when using the original formula: HOMA- IR = fasting insulin (mU/L) x fasting plasma (mg/dL) / 405. In this highly regulated mechanism, insulin resistance occurs when a higher than normal insulin concentration is required to obtain a quantitatively normal response in target tissues, and, among other parameters, HOMA-IR values start to increase [19]. During this initial compensatory phase of early insulin resistance, higher HOMA-IR values, among other insulin resistance surrogates such as the triglyceride-glucose index [20], are associated with markers of ectopic fat accumulation and metabolic syndrome, including elevated circulating triglycerides, adipose tissue dysfunction, increased secretion of free fatty acids (FFAs) and pro-inflammatory adipokines, and elevated liver production of very-low-density lipoprotein (VLDL) particles in the presence of visceral fat and hepatic fat [21]. Additionally, HOMA-IR levels are elevated in patients with heart failure, a condition often accompanied by comorbidities such as obesity and hypertension [22], and have been identified as predictor of adverse clinical events in non-diabetic individuals experiencing decompensated heart failure [23]. In this regard, it is not surprising that in our study, higher HOMA-IR values were associated with a cluster of unfavorable anthropometric, metabolic, and inflammatory markers (p < 0.001, Table 1) in individuals with low-to-moderate and high CVD risk (Supplementary Tables 1 and 2).

### Association of HOMA-IR with subclinical atherosclerosis

Our analysis establishes an association between higher HOMA-IR values and the multiterritorial extent of SA and CACS after adjusting for key CVRFs and HbA1c (Fig. 2A). Similar results have been previously documented in other cohorts and with different imaging methodologies in adults, children, and adolescents, although these analyses used less robust markers of SA, like carotid intima-media thickness (cIMT) [24]. Studies of populations without type 2 diabetes have reported associations of HOMA-IR with CAC prevalence and progression for some ethnicities [25, 26] and with the severity of multivessel coronary artery disease [27]. Our results, obtained with 2DVUS and CACS imaging techniques and including assessment of global plaque volume by 3DVUS, support previous findings in individuals with less advanced disease status (HbA1c < 6.0% (< 42 mmol/mol) and suggest that early insulin resistance may be an indicator of higher risk for the development of SA, independently of other CVRFs and HbA1c. These findings highlight the importance of early screening to identify at-risk individuals before clinical CVD events occur and to reduce the risk of future type 2 diabetes.

The relationship between HOMA-IR and SA described here was particularly striking for the coronary territory. In the reference HOMA-IR group (< 2), only 14.9% had a CACS > 0; in other words, 85.1% were free of coronary artery calcium (Fig. 2D). The proportion of participants with CACS increased with the insulin-resistance category, to 25.6% for HOMA-IR = 2 to 3 and 39.3% for HOMA-IR ≥ 3. When further studying the participant subgroup with the highest values of HOMA-IR ≥ 4 (n = 49), the proportion with CACS > 0 increased to 49% (more than 3 times the rate in the reference HOMA-IR group). It is important to note that all the study participants are asymptomatic individuals considered to be in primary prevention, with mainly low-to-moderate SCORE2 risk estimates. HOMA-IR thus appears to identify the risk of coronary artery calcification in this overtly disease-free population, even after adjusting for other CVRFs and HbA1c. It is unclear why the association between HOMA-IR and SA was only observed in individuals with a low-to-moderate SCORE2 risk estimate and not in those with high CVD risk. It may be that SCORE2, designed to predict the risk of clinical events, is less able to predict SA. The fact that a notable occurrence of multi-territorial SA within the low-to-moderate risk group is observed could be attributed to the comprehensive examination of various territories, including vascular areas more susceptible to disease, such as the iliofemoral arteries, which were not explored in earlier studies. Another possibility is that the high CVD-risk subgroup had a higher prevalence of traditional CVRFs that heavily influenced the SCORE2 equations or that the sample size for this risk category was too small, leaving our study underpowered to detect differences in this subgroup. These results emphasize the importance of exploring the relationship between the early onset of insulin resistance and SA, especially in individuals with low-to-moderate CVD risk, who often receive less attention in clinical practice but who may have a substantial burden of atherosclerotic disease.

### Potential of HOMA-IR as a marker of metabolic syndrome and NAFLD

In clinical practice, HOMA-IR is often used as an indicator of metabolic syndrome, a condition characterized by a clustering of risk factors indicative of poor metabolic health but for which clinical utility beyond its individual components is still disputed [28]. In our study population, the HOMA-IR category (< 2, 2 to 3, and ≥ 3) showed a direct correlation with the prevalence of metabolic syndrome (3.0%, 27.1%, and 49.3%) (Table 1) and hepatic steatosis (0.8%, 21.9%, and 59.3%) (p < 0.001, Supplementary Tables 3 and Supplementary Fig. 4). An exploratory analysis showed that individuals meeting NAFLD diagnostic criteria had increased odds of multiterritorial SA (OR = 1.45 (95%CI: 1.05 to 2.00), p = 0.023) and CACS (OR = 2.32 (95%CI: 1.59 to 3.40), p < 0.001) independently of all other CVRFs evaluated, including HOMA-IR. Although all these parameters are highly related and share common pathophysiological pathways, in clinical practice, HOMA-IR offers the potential to detect and monitor a common root cause of both conditions - insulin resistance and insulin hypersecretion - with a convenient measure obtained from a blood test. HOMA-IR could be particularly useful for the early detection of NAFLD, a condition whose prevalence is projected to increase in the coming decades, often goes undetected until its later stages, and for which there is currently no approved effective treatment [29].

### Measuring HOMA-IR in individuals with low-to-moderate CVD risk: an opportunity to initiate prevention

Almost 60% of PESA participants have SA despite being classified as low CVD risk in the baseline evaluation [13]. In this substudy of individuals with HbA1c < 6.0% (< 42 mmol/mol), measuring insulin resistance with the HOMA-IR ratio added clinical value by detecting individuals at higher risk of developing SA, particularly among the subgroup with a low-to-moderate SCORE2 risk estimate.

Beginning intervention at an early disease stage is typically less costly, less risky, and more effective. Identifying individuals in the early stages of insulin resistance is likely to increase the potential to significantly reduce the occurrence of cardiovascular events over time. However, it is important to note that further research is needed to establish this effect with greater certainty. Furthermore, repeated measures of HOMA-IR could be used to monitor changes in individual risk and health status. For instance, the inclusion of fasting insulin in addition to fasting glucose or HbA1c in asymptomatic individuals could potentially identify individuals at risk of type 2 diabetes years before disease initiation. Although there are currently no medications specifically approved to treat insulin resistance [30], HOMA-IR provides an effective way to monitor lifestyle interventions designed to improve insulin sensitivity, such as weight loss, exercise, and diet replacement interventions. In addition, our results indicate that HOMA-IR monitoring may help to mitigate the risk of developing metabolic syndrome and NAFLD, as well as the overall SA disease burden.

While the HOMA-IR index has been widely utilized in epidemiological settings, we acknowledge that the simplicity and cost-effectiveness of measuring HOMA-IR may be subject to variability, particularly given the non-standardized nature of insulin determinations and variations among different populations. We recognize that its suitability in clinical practice is a topic of debate, and further considerations are warranted, especially considering the projected increase in CVRFs and CVD prevalence [31].

### Study limitations

Several study limitations need to be acknowledged. First, this was a cross-sectional study, and the association detected between HOMA-IR and SA, therefore, cannot be considered causal. In addition, any residual confounding resulting from unmeasured or inadequately controlled variables cannot be ruled out. It should be noted that the PESA study cohort is composed of individuals from a specific occupation and living in Spain, and therefore, caution should be exercised when generalizing the results. The prevalence of multiterritorial disease in this cohort is relatively high among subjects considered at low-to-moderate risk. While the SCORE2 scale was designed to evaluate the risk of CV events stemming from atherosclerosis, it was not intended to estimate the presence of SA. Our study suggests the added value of imaging for diagnosis and prevention beyond traditional CV risk scores. Furthermore, it underscores the potential to identify easily measurable markers, as demonstrated in this study with early insulin resistance markers, to predict the presence of SA. Lastly, further studies are needed to define the relationship between the HOMA-IR ratio and other CVRFs, including genetics, lifestyle, and environmental factors. This could lead to a more comprehensive understanding of the underlying mechanisms of CVD and facilitate the development of more effective prevention strategies.

## Conclusions

The results of this cross-sectional study suggest that assessment of the HOMA-IR ratio in populations without prediabetes or type 2 diabetes can provide valuable information for identifying individuals at a higher risk of developing subclinical atherosclerosis. Through a simple and economical blood test, HOMA-IR informs on the onset of insulin resistance and may help to identify the need for earlier implementation of prevention strategies. These findings highlight the importance of including the HOMA-IR ratio in the assessment of cardiovascular risk and underscore the potential benefits of early intervention in primary prevention.

### Electronic supplementary material

Below is the link to the electronic supplementary material.

Supplementary Material 1

## Data Availability

The datasets used and/or analyzed during the current study are available from the corresponding author upon reasonable request.

## List of abbreviations

CVD: Cardiovascular disease
HbA1c: Glycated hemoglobin
SA: Subclinical atherosclerosis
CVRFs: Cardiovascular risk factors
HOMA-IR: Homeostatic Model Assessment of Insulin Resistance
PESA: Progression of Early Subclinical Atherosclerosis
SBP: Systolic blood pressure
DBP: Diastolic blood pressure
HDL-C: High-density lipoprotein cholesterol
LDL-C: Low-density lipoprotein cholesterol
TyG: Triglyceride to glucose
NAFLD: Non-alcoholic fatty liver disease
ALT: Alanine transaminase
ALT: Aspartate transaminase
2DVUS: 2-dimensional vascular ultrasound
CACS: Coronary artery calcium score
3DVUS: 3-dimensional vascular ultrasound
SD: Standard deviation
BMI: Body mass index
hs-CRP: high-sensitivity C-reactive protein
FFAs: Free fatty acids
VLDL: Very-low-density lipoprotein
MAFLD: Metabolic (dysfunction)-associated fatty liver disease
cIMT: Carotid intima-media thickness
