# Supplementary material for: Early insulin resistance in normoglycemic low-risk individuals is associated with subclinical atherosclerosis
Source: Cardiovasc Diabetol. 2023 Dec 19;22:350. doi: 10.1186/s12933-023-02090-1 (PMC10731750; doi:10.1186/s12933-023-02090-1)
Supplement: Supplementary file 1 — Supplementary Material 1 [file 12933_2023_2090_MOESM1_ESM.docx]

**SUPPLEMENTAL MATERIAL**

[**SUPPLEMENTARY TABLES** 2](#_Toc152249856)

[**SUPPLEMENTARY FIGURES** 9](#_Toc152249857)

# **SUPPLEMENTARY TABLES**

**Supplementary Table 1.** Study Population Clinical Characteristics for Participants with Low-to-moderate SCORE2 Risk (N = 2,830), stratified by HOMA-IR Category

| **LOW-TO-MODERATE SCORE2 RISK** | **Total Population**  **(N = 2,830)** | **HOMA-IR < 2**  **(n=2,522)** | **HOMA-IR 2 to <3**  **(n=237)** | **HOMA-IR ≥ 3**  **(n=71)** | **P Value** |
| --- | --- | --- | --- | --- | --- |
| **Demographic**  Age, years  Sex, male  Race/Ethnicity, Caucasian | 45.3 ± 4.3  1,441 (50.9)  2,826 (99.9) | 45.2 ± 4.3  1,207 (47.9)  2,518 (99.8) | 46.4 ± 4.6  179 (75.5)  237 (100) | 46.9 ± 4.9  55 (77.5)  71 (100) | <0.001^*^^  <0.001^*^^  0.901 |
| **Risk Factors**  Hypertension  Dyslipidemia  Smoking  Sedentary lifestyle  Family History of CV Disease  Metabolic syndrome | 205 (7.2)  860 (30.4)  336 (11.9)  2,757 (98.4)  437 (15.4)  111 (3.9) | 141 (5.6)  686 (27.2)  327 (13.0)  2,457 (98.4)  375 (14.9)  41 (1.6) | 42 (17.7)  138 (58.2)  7 (3.0)  230 (98.3)  43 (18.1)  48 (20.2) | 22 (31.0)  36 (50.7)  2 (2.8)  70 (98.6)  19 (26.8)  22 (31.0) | <0.001^*^^  <0.001^*^^  <0.001^*^^  0.902  0.001^^^  <0.001^*^^ |
| **Measured cardiovascular risk and health factors**  SBP, mmHg  DBP, mmHg  Total Cholesterol, mg/dL  HDL-C, mg/dL  LDL-C, mg/dL  Non-HDL-C, mg/dL  Triglycerides, mg/dL  BMI, kg/m2  Waist, cm  Basal Insulin, mU/L  Basal glucosa, mg/dl  HbA1c, %  Ratio Total-Cholesterol/HDL  Ratio Total-Cholesterol/HDL  TyG index  Mod/vig activity, min/week | 113.3 ± 11.5  70.5 ± 8.6  195.3 ± 30.7  52.0 ± 12.2  127.1 ± 27.0  143.3 ±29.8  70 [55-95]  25.3 ± 3.8  86.2 ± 11.3  4.6 [3.4-6.5]  87 [82-93]  5.4 [5.1-5.6]  3.8 [3.2-4.5]  1.4 [1.0-2.1]  4.4 [4.2-4.5]  240 [159-347] | 112.4 ± 11.2  69.7 ± 8.3  194.4 ± 30.5  52.9 ± 12.1  126.2 ± 27.0  141.5 ± 29.3  68 [53-89]  24.8 ± 3.2  84.6 ± 10.4  4.3 [3.2-5.7]  86 [82-91]  5.3 [5.1-5.5]  3.7 [3.1-4.4]  1.3 [0.9-1.9]  4.3 [4.2-4.5]  241 [161-348] | 120.7 ± 10.6  76.8 ± 8.3  201.6 ± 31.0  43.9 ± 9.5  134.7 ± 25.8  157.7 ± 28.3  106 [78-134]  29.1 ± 3.2  98.1 ± 9.8  10.1 [9.3-11.0]  95 [90-100]  5.4 [5.2-5.7]  4.5 [4.0-5.4]  2.4 [1.7-3.5]  4.6 [4.5-4.7]  230 [150-328] | 121.5 ± 10.4  77.2 ± 8.8  206.2 ± 32.5  43.6 ± 9.7  135.5 ± 26.5  162.5 ± 31.0  125 [88-173]  31.1 ± 3.8  102.9 ± 9.9  14.8 [13.5-17.9]  97 [93-101]  5.5 [5.3-5.7]  4.9 [4.0-5.4]  3.1 [1.9-4.3]  4.7 [4.5-4.9]  259 [148-394] | <0.001^*^^  <0.001^*^^  <0.001^*^^  <0.001^*^^  <0.001^*^^  <0.001^*^^  <0.001^*^^  <0.001^*^^  <0.001^*^^  <0.001^*^^  <0.001^*^^  <0.001^*^^  <0.001^*^^  <0.001^*^^  <0.001^*^^  0.017 |
| **Inflammatory markers**  hs-CRP, mg/dL  Ferritin, ng/mL  1-hour ESR, mm  Fibrinogen, mg/dL | 0.08 [0.04-0.16]  47.2 [21.9-101.3]  6 [4-8]  257.5 [231.4-288.2] | 0.08 [0.04-0.15]  44.8 [21.1-93.0]  6 [4-8]  256.6 [231.2-287.4] | 0.14 [0.08-0.25]  105.3 [45.2-191.3]  5 [4-8]  261.1 [232.9-292.3] | 0.15 [0.09-0.30]  104.2 [33.4-193.1]  7 [4-9]  271.3 [244.5-304.6] | <0.001^*^^  <0.001^*^^  0.696  <0.001^^^ |
| **Risk scores**  SCORE2, %  Framingham Risk Score 10y, %  Framingham Risk Score 30y, %  Regicor Risk Score, % | 1.65 [1.03-2.24]  4.40 [2.91-6.37]  11.30 [6.96-16.46]  1.26 [0.78-1.87] | 1.57 [0.98-2.15]  4.17 [2.81-6.02]  10.6 [6.59-15.59]  1.20 [0.72-1.75] | 2.17 [1.71-2.95]  6.38 [5.01-8.72]  16.98 [12.69-21.61]  1.96 [1.40-2.62] | 2.41 [1.74-3.28]  7.13 [4.77-11.23]  18.79 [13.35-25.22]  2.21 [1.47-2.97] | <0.001^*^^  <0.001^*^^  <0.001^*^^  <0.001^*^^ |
| **Liver measurements**  ALT, U/L  AST, U/L  NAFLD score  Liver fat, %  Liver steatosis (≥5% liver fat) | 17 [13-24]  18 [15-21]  -2.44 [-2.82; -1.95]  1.52 [1.20-2.06]  80 (2.8) | 17 [12-22]  17 [15-21]  -2.53 [-2.85; -2.13]  1.44 [1.16-1.84]  9 (0.4) | 27 [19-36]  20 [17-24]  -1.14 [-1.49; -0.58]  3.08 [2.50-4.08]  39 (16.5) | 29 [19-38]  19 [17-25]  -0.11 [-0.72; 0.84]  4.39 [3.09-6.19]  32 (45.1) | <0.001^*^^  <0.001^*^^  <0.001^*^^  <0.001^*^^  <0.001^*^^ |

Values are mean ± SD, n (%), or median [first quartile, third quartile]. Indicated p-value derived from trend tests among HOMA-IR categories. * indicates statistically significant differences (p<0.025) between HOMA-IR < 2 and HOMA-IR 2 to < 3 groups. ^ indicates statistically significant differences (p<0.025) between HOMA-IR < 2 and HOMA-IR ≥3 groups. HOMA-IR = homeostatic model assessment of insulin resistance; CV = cardiovascular; SBP = systolic blood pressure; DBP = diastolic blood pressure; HDL-C = high-density lipoprotein cholesterol; LDL-C = low-density lipoprotein cholesterol; BMI = body mass index; HbA1c = glycated hemoglobin; TyG = triglyceride to glucose; Mod/Vig activity min/week = moderate to vigorous minutes of physical activity per week; hs-CRP *=* high-sensitivity C-reactive protein*;* 1-hour ESR = erythrocyte sedimentation rate, SCORE2 = systematic coronary risk estimation 2; ALT = alanine transaminase; AST = aspartate transaminase; NAFLD = non-alcoholic fatty liver disease.

**Supplementary Table 2.** Study Population Clinical Characteristics for Participants with High SCORE2 Risk (N = 911), stratified by HOMA-IR Category

| **HIGH SCORE2 RISK** | **Total Population**  **(N = 911)** | **HOMA-IR < 2**  **(n=659)** | **HOMA-IR 2 to < 3**  **(n=173)** | **HOMA-IR ≥ 3**  **(n=79)** | **P Value** |
| --- | --- | --- | --- | --- | --- |
| **Demographic**  Age, years  Sex, male  Race/Ethnicity, caucasian | 46.3 ± 3.7  854 (93.7)  910 (99.9) | 46.2 ± 3.7  608 (92.3)  658 (99.8) | 46.4 ± 3.8  170 (98.3)  173 (100) | 46.9 ± 3.3  76 (96.2)  79 (100) | 0.082  0.016^*^  0.766 |
| **Risk Factors**  Hypertension  Dyslipidemia  Smoking  Sedentary lifestyle  Family History of CV Disease  Metabolic syndrome | 184 (20.2)  608 (66.7)  407 (45.6)  885 (98.9)  148 (16.2)  168 (18.4) | 103 (15.6)  410 (62.2)  311 (48.1)  642 (99.2)  99 (15.0)  53 (8.0) | 53 (30.6)  133 (76.9)  75 (45.2)  165 (97.1)  36 (20.8)  63 (36.4) | 28 (35.4)  65 (82.3)  21 (26.6)  78 (100)  13 (16.5)  52 (65.8) | <0.001^*^^  <0.001^*^^  <0.001^^^  0.745^*^  0.667  <0.001^*^^ |
| **Measured cardiovascular risk and health factors**  SBP, mmHg  DBP, mmHg  Total Cholesterol, mg/dL  HDL-C, mg/dL  LDL-C, mg/dL  Non-HDL-C, mg/dL  Triglycerides, mg/dL  BMI, kg/m2  Waist, cm  Basal Insulin, mU/L  Basal glucosa, mg/dL  HbA1c, %  Ratio Total-Cholesterol/HDL  Ratio Total-Cholesterol/HDL  TyG index  Mod/vig activity, min/week | 123.2 ± 12.4  77.0 ± 9.8  213.9 ± 34.6  41.8 ± 8.5  146.7 ± 30.5  172.1 ±33.6  108 [83-147]  27.6 ± 3.3  95.5 ± 9.2  6.5 [4.4-9.0]  92 [87-98]  5.4 [5.2-5.6]  5.1 [4.4-6.1]  2.6 [1.9-3.8]  4.6 [4.5-4.8]  247 [164-348] | 121.8 ± 11.8  75.6 ± 9.5  212.0 ± 34.0  42.9 ± 8.3  146.2 ± 30.0  169.1 ± 32.9  102 [78-137]  26.7 ± 2.8  93.0 ± 8.3  5.4 [3.9-6.8]  90 [86-95]  5.4 [5.2-5.6]  4.9 [4.3-5.8]  2.5 [1.8-3.3]  4.6 [4.4-4.7]  257 [170-356] | 126.1 ± 13.7  80.0 ± 10.1  219.8 ± 37.6  40.1 ± 8.9  150.2 ± 33.3  179.7 ± 36.1  133 [97-171]  29.5 ± 3.2  101.3 ± 7.8  10 [9.2-11]  95 [91-101]  5.4 [5.2-5.7]  5.5 [4.6-6.5]  3.4 [2.3-4.5]  4.7 [4.6-4.9]  237 [154-338] | 128.7 ± 11.9  81.7 ± 9.2  217.6 ± 31.4  37.1 ± 6.2  144.0 ± 28.1  180.5 ± 29.8  155 [107-233]  30.8 ± 3.3  103.9 ± 8.7  15 [13.2-17.6]  101 [96-106]  5.6 [5.4-5.8]  5.9 [5.1-6.7]  4.1 [2.6-6.6]  4.8 [4.6-5.1]  191 [139-303] | <0.001^*^^  <0.001^*^^  <0.001^*^  <0.001^*^^  0.161  <0.001^*^^  <0.001^*^^  <0.001^*^^  <0.001^*^^  <0.001^*^^  <0.001^*^^  <0.001^^^  <0.001^*^^  <0.001^*^^  <0.001^*^^  <0.001^^^ |
| **Inflammatory markers**  hs-CRP, mg/dL  Ferritin, ng/mL  1-hour ESR, mm  Fibrinogen, mg/dL | 0.12 [0.07-0.23]  141.5 [77.5-225.6]  5 [4-7]  261.3 [233.2-291.3] | 0.11 [0.06-0.20]  131.42 [73.8-206.5]  5 [4-7]  259.8 [231.7-288.9] | 0.16 [0.10-0.29]  170.2 [109.6-268.3]  5 [4-7]  263.6 [235-302.2] | 0.17 [0.10-0.31]  168.3 [100.8-274.1]  6 [4-9]  266.7 [251.9-291.3] | 0.005^*^^  0.002^*^  0.018^^^  0.040 |
| **Risk scores**  SCORE2, %  Framingham Risk Score 10y, %  Framingham Risk Score 30y, %  Regicor Risk Score, % | 3.52 [2.89-4.79]  10.84 [8.52-15.04]  26.80 [21.76-35.09]  3.04 [2.39-4.10] | 3.44 [2.87-4.57]  10.16 [8.24-14.29]  25.46 [21.04-33.19]  2.94 [2.31-3.91] | 3.85 [2.92-5.42]  11.88 [8.93-18.22]  31.24 [23.21-40.48]  3.28 [2.57-4.94] | 3.78 [3.03-5.00]  12.51 [9.98-17.22]  31.19 [25.60-40.14]  3.51 [2.56-4.56] | <0.001^*^  <0.001^*^^  <0.001^*^^  <0.001^*^^ |
| **Liver measurements**  ALT, U/L  AST, U/L  NAFLD score  Liver fat, %  Liver steatosis (≥5% liver fat) | 24 [18-32]  20 [17-23]  -1.86 [-2.36; -1.04]  2.19 [1.60-3.32]  124 (13.6) | 22 [17-29]  19 [17-23]  -2.09 [-2.49; -1.71]  1.89 [1.46-2.39]  16 (2.4) | 29 [21-39]  21 [18-25]  -0.85 [-1.31; -0.21]  3.76 [2.82-5.27]  51 (29.5) | 32 [24-43]  21 [17-26]  0.70 [-0.01;1.24]  6.84 [3.90-8.34]  57 (72.1) | <0.001^*^^  0.001^*^^  <0.001^*^^  <0.001^*^^  <0.001^*^^ |

Values are mean ± SD, n (%), or median [first quartile, third quartile]. Indicated p-value derived from trend tests among HOMA-IR categories. * indicates statistically significant differences (p<0.025) between HOMA-IR < 2 and HOMA-IR 2 to < 3 groups. ^ indicates statistically significant differences (p<0.025) between HOMA-IR < 2 and HOMA-IR ≥3 groups. HOMA-IR = homeostatic model assessment of insulin resistance; CV = cardiovascular; SBP = systolic blood pressure; DBP = diastolic blood pressure; HDL-C = high-density lipoprotein cholesterol; LDL-C = low-density lipoprotein cholesterol; BMI = body mass index; HbA1c = glycated hemoglobin; TyG = triglyceride to glucose; Mod/Vig activity min/week = moderate to vigorous minutes of physical activity per week; hs-CRP = high-sensitivity C-reactive protein; 1-hour ESR = erythrocyte sedimentation rate, SCORE2 = systematic coronary risk estimation 2; ALT = alanine transaminase; AST = aspartate transaminase; NAFLD = non-alcoholic fatty liver disease.

**Supplementary Table 3.** Study Population Liver Measurements (N = 3,741) Stratified by HOMA-IR Category

|  | **Total Population**  **(N = 3,741)** | **HOMA-IR < 2**  **(n=3,181)** | **HOMA-IR 2 to <3**  **(n=410)** | **HOMA-IR ≥ 3**  **(n=150)** | **P Value** |
| --- | --- | --- | --- | --- | --- |
| **Liver measurements**  ALT, U/L  AST, U/L  NAFLD score  Liver fat, %  Liver steatosis (≥5% liver fat) | 19 [14-26]  18 [15-22]  -2.33 [-2.73; -1.75]  1.64 [1.25-2.31]  204 (5.4) | 18 [13-24]  18 [15-21]  -2.45 [-2.80; -2.03]  1.51 [1.20-1.97]  25 (0.8) | 28 [20-37]  20 [17-24]  -1.04 [-1.44; -0.33]  3.22 [2.58-4.70]  90 (21.9) | 31 [21-41]  20 [17-25]  0.46 [-0.60; 1.04]  5.73 [3.58-7.64]  89 (59.3) | <0.001^*^^  <0.001^*^^  <0.001^*^^  <0.001^*^^  <0.001^*^^ |

Values are mean ± SD, n (%), or median [first quartile, third quartile]. Indicated p-value derived from trend tests among HOMA-IR categories. * indicates statistically significant differences (p<0.025) between HOMA-IR < 2 and HOMA-IR 2 to < 3 groups. ^ indicates statistically significant differences (p<0.025) between HOMA-IR < 2 and HOMA-IR ≥3 groups. HOMA-IR = homeostatic model assessment of insulin resistance; ALT = alanine transaminase; AST = aspartate transaminase; NAFLD = non-alcoholic fatty liver disease.

# **SUPPLEMENTARY FIGURES**

**Supplementary Figure 1.** HOMA-IR distribution among study participants

HOMA-IR = homeostatic model assessment of insulin resistance

**Supplementary Figure 2.** HOMA-IR Categories and Association with Multi-territorial Extent of Subclinical Atherosclerosis in Different Vascular Territories for individuals with HbA1c <5.5% (below the median).

| **Panel A**  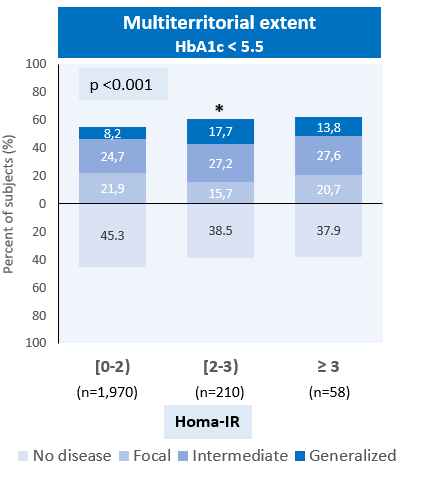 | **Panel B**  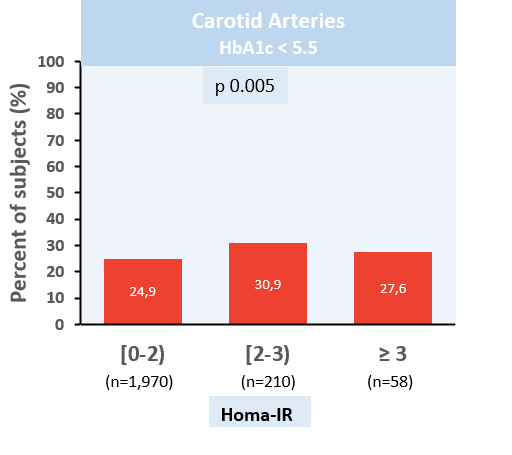 |
| --- | --- |
| **Panel C**  **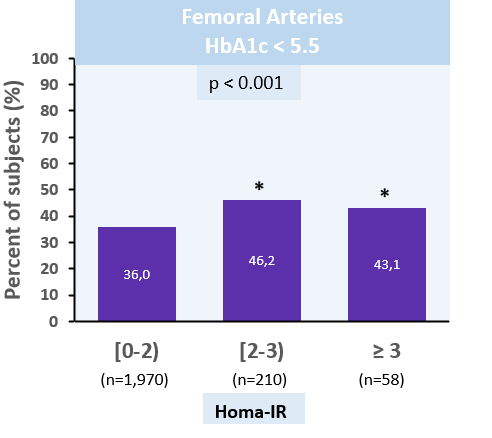** | **Panel D**  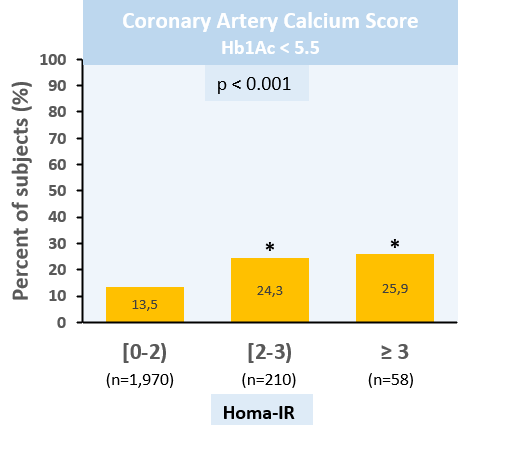 |

**(A)** Multiterritorial extent of subclinical atherosclerosis (SA) assessed by 2-dimensional vascular ultrasound and non-contrast coronary [computed tomography](https://www.sciencedirect.com/topics/nursing-and-health-professions/computer-assisted-tomography), stratified by HOMA-IR category for individuals with HbA1c < 5.5% (below the median). Multiterritorial SA extent for this figure is defined by combining data from both [imaging techniques](https://www.sciencedirect.com/topics/medicine-and-dentistry/imaging-technique) to classify individuals as having no disease (0 vascular sites affected) or having focal (1 site), intermediate (2 to 3 sites), or generalized atherosclerosis (4 to 6 sites). **(B-D)** Presence of SA in different vascular territories stratified by HOMA-IR category. * indicates statistically significant differences (p<0.025) as compared to the lowest HOMA-IR category (HOMA-IR <2, reference group). HbA1c = glycated hemoglobin, HOMA-IR = homeostatic model assessment of insulin resistance.

**Supplementary Figure 3.** HOMA-IR Categories and Association with Multi-territorial Extent of Subclinical Atherosclerosis in Different Vascular Territories for individuals with HbA1c ≥5.5% (above the median).

| **Panel A**  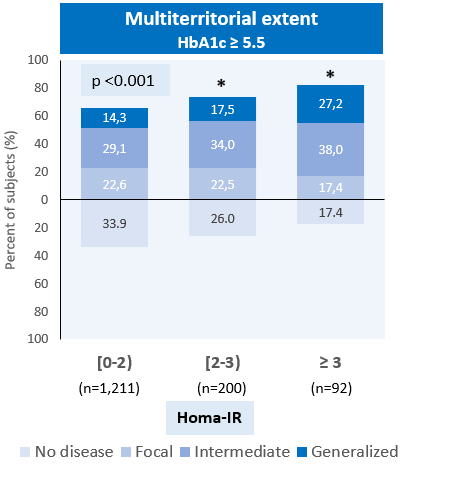 | **Panel B**  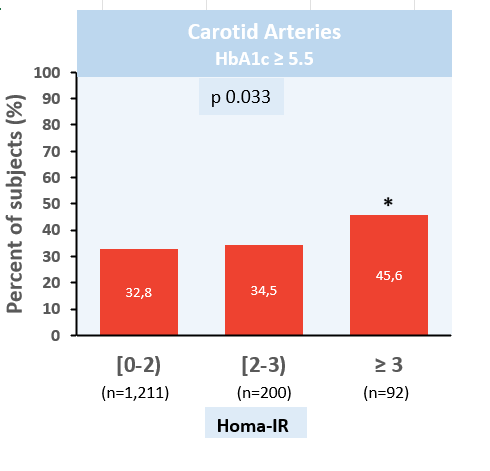 |
| --- | --- |
| **Panel C**  **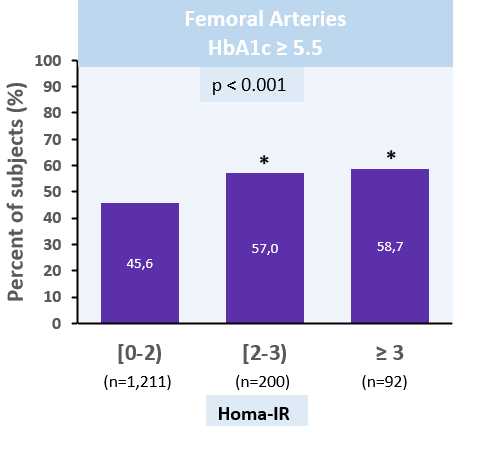** | **Panel D**  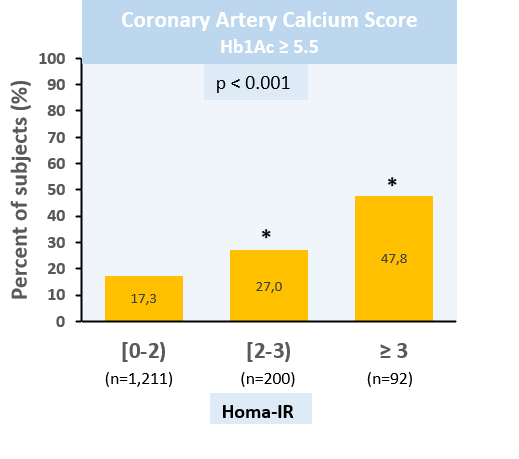 |

**(A)** Multiterritorial extent of subclinical atherosclerosis (SA) assessed by 2-dimensional vascular ultrasound and non-contrast coronary [computed tomography](https://www.sciencedirect.com/topics/nursing-and-health-professions/computer-assisted-tomography), stratified by HOMA-IR category for individuals with HbA1c ≥ 5.5% (above the median). Multiterritorial SA extent for this figure is defined by combining data from both [imaging techniques](https://www.sciencedirect.com/topics/medicine-and-dentistry/imaging-technique) to classify individuals as having no disease (0 vascular sites affected) or having focal (1 site), intermediate (2 to 3 sites), or generalized atherosclerosis (4 to 6 sites). **(B-D)** Presence of SA in different vascular territories stratified by HOMA-IR category. * indicates statistically significant differences (p<0.025) as compared to the lowest HOMA-IR category (HOMA-IR <2, reference group). HbA1c = glycated hemoglobin, HOMA-IR = homeostatic model assessment of insulin resistance.

**Supplementary Figure 4.** Association of HOMA-IR with Metabolic Syndrome and Hepatic Steatosis

**Panel A**

**Panel B**

**(A)** Proportion of study participants with metabolic syndrome across HOMA-IR categories. Metabolic syndrome was defined according to the modified ATP III criteria. **(B)** Proportion of study participants with hepatic steatosis across across HOMA-IR categories. Hepatic steatosis was defined as intrahepatic fat ≥ 5% liver weight, estimated with the non-alcoholic fatty liver disease (NAFLD) score from routinely available clinical data and serological markers.
